# Supplementary material for: Experimental, Spectroscopic, and Computational Insights into the Reactivity of “Methanal” with 2-Naphthylamines
Source: Molecules. 2023 Feb 6;28(4):1549. doi: 10.3390/molecules28041549 (PMC9964406; doi:10.3390/molecules28041549)

Electronic Supporting information

Experimental, Spectroscopic, and Computational Insights into the Reactivity of "Methanal" with 2-Naphthylamines

Martin Havlík [1], Tereza Navrátilová [1], Michaela Drozdová [1], Ameneh Tatar [1], Priscila A. Lanza [2], Diego Dusso [2,3], Elizabeth Laura Moyano [3], Carlos A. Chesta [4], Domingo Mariano A. Vera [2,*] and Bohumil Dolenský [1,*]

[1] Department of Analytical Chemistry, University of Chemistry and Technology Prague, Technická 5, 166 28 Praha, Czech Republic

[2] QUIAMM-INBIOTEC, Department of Chemistry, Facultad de Ciencias Exactas y Naturales, Universidad Nacional de Mar del Plata, B7602AYL, Mar del Plata, Argentina

[3] INFIQC, Department of Organic Chemistry, Facultad de Ciencias Químicas, Universidad Nacional de Córdoba, 5000, Córdoba, Argentina

[4] Instituto de Investigaciones en Tecnologías Energéticas y Materiales Avanzados (IITEMA), Universidad Nacional de Río Cuarto (UNRC) and Consejo Nacional de Investigaciones Científicas y Tecnológicas (CONICET), Campus Universitario, (5800) Río Cuarto, Argentina

* Correspondence: dmavera@yahoo.com (D.M.A.V.); dolenskb@vscht.cz (B.D.)

S2	**Table S1.** Computational details on the stationary points discussed
S3	**Scheme S1** and **Scheme S2.** Comparison of the thermochemistry obtained including dispersion effects (CAM-B3LYP-GD3BJ) and without dispersion (CAM-B3LYP)
S4 – S7	3D views of stationary points **8b**, **TS-i2-6a**, **6a**, **7a**, **i3b**, **i3b$_{tau}$**, **TS-9-6b$_{tau}$**, **TS-i3-3a**, and **3b**
S8	^{1}H and ^{13}C NMR spectra of pure quinazoline **6a**
S9	^{1}H and ^{13}C NMR spectra of pure bisquinazoline **16a**
S10	^{1}H NMR spectra of crude **8b** in DMSO-d_6 at various temperatures
S11	^{1}H-^{1}H DQF-COSY 2D NMR spectrum of **1**, **2b**, **8b**, **9b**, and **20b** mixture
S12	^{1}H-^{13}C HSQC and HMBC 2D NMR spectra of **1**, **2b**, **8b**, **9b**, and **20b** mixture
S13	^{1}H NMR spectrum of formalin in DMSO-d_6 at 25°C
S14	^{1}H-^{13}C HSQC and HMBC 2D NMR spectra of formalin

Table S1. Computational details on the stationary points discussed. All energies in Hartree.

Compound	G° CAM-B3LYP-GD3JB	G° CAM-B3LYP
H_2CO	-114.491640	-114.489964
H_2O	-1.174208	-76.432964
H_2	-1.174208	-1.172689
**8a**	-920.073868	-920.0287250
**8b**	-1375.747036	-1375.690427
**9a**	-479.063041	-479.042615
**9b**	-706.898231	-706.872007
**2a**	-441.005101	-440.986296
**2b**	-668.842031	-668.817432
**TS-2-i1-a**	-555.451454	-555.427415
**TS-2-i1-b**	-783.285908	-783.256058
**i1a**	-555.499702	-555.476212
**i1b**	-783.336901	-783.307603
**TS-9+i1-i2-a**	-1034.522700	-1034.471547
**TS-9+i1-i2-b**	-1490.196533	-1490.133616
**i2a**	-1034.564492	-1034.511024
**i2b**	-1490.236841	-1490.171939
**TS-i2-6-a**	-1034.444599	-1034.390292
**TS-i2-6-b**	-1490.114481	-1490.048534
**6a**	-958.152910	-958.104100
**6b**	-1413.827028	-1413.765232
**TS-9-6$_{tau}$-a**	-958.041781	-957.989249
**TS-9-6$_{tau}$-b**	-1413.711896	-1413.647698
**6$_{tau}$a**	-958.128114	-958.104138
**6$_{tau}$b**	-1413.798931	-1413.765232
**7a**	-956.974997	-956.927692
**7b**	-1412.645328	-1412.586375
**TS-6-i3-a**	-1072.567777	-1072.535994
**TS-6-i3-b**	-1528.263784	-1528.195406
**i3a**	-1072.592746	-1072.536200
**i3b**	-1528.264687	-1528.196409
**i3$_{tau}$a**	-1072.646637	-1072.591200
**i3$_{tau}$b**	-1528.319334	-1528.252168
**TS-i3$_{tau}$-3-a**	-1072.576991	-1072.520054
**TS-i3$_{tau}$-3-b**	-1528.243061	-1528.173841
**3a**	-996.237418	-996.182569
**3b**	-1451.908422	-1451.841888
N-methylnaphthylamine (**a**)	-480.270614	-480.249252
N-methylnaphthylamine (**b**)	-708.108099	-708.080945

Comparison of the thermochemistry obtained

including dispersion effects (CAM-B3LYP-GD3BJ) and without dispersion (CAM-B3LYP)

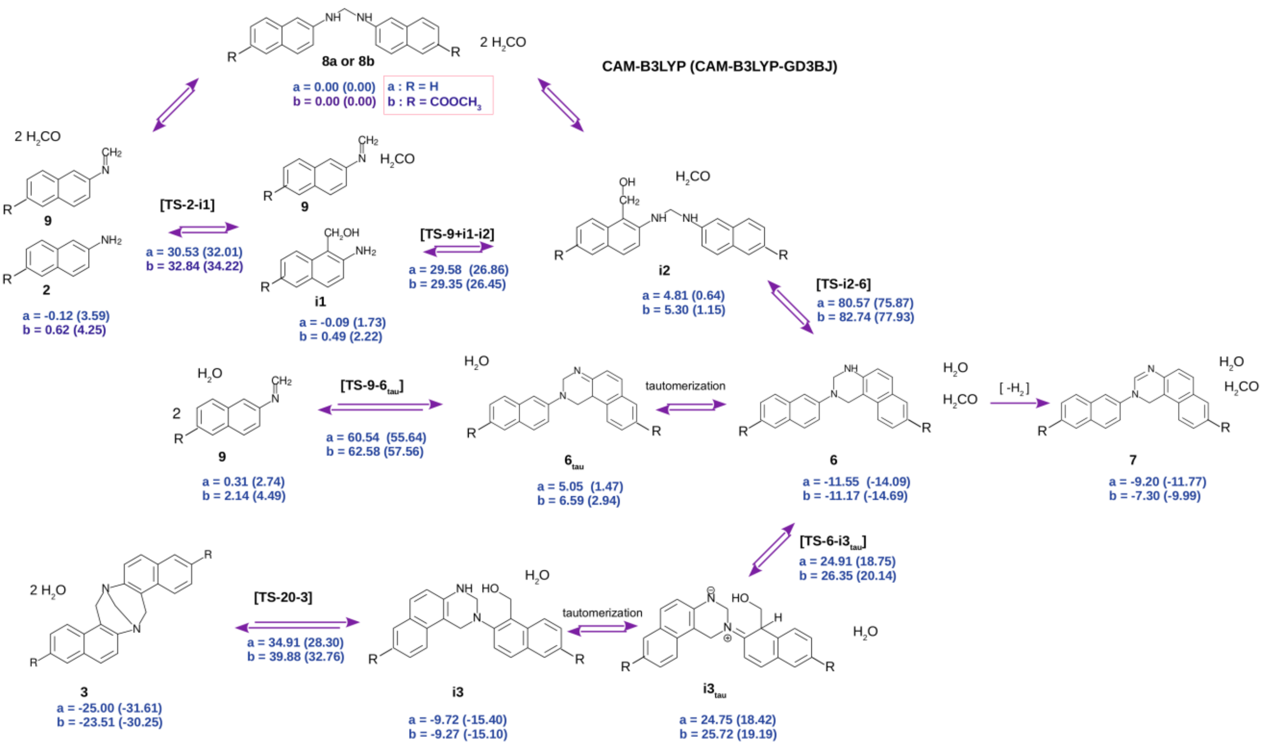

Scheme S1. Comparison between the thermochemistry with uncorrected CAM-B3LYP and with CAM-B3LYP-GD3BJ (in parenthesis). As main text Scheme 8.

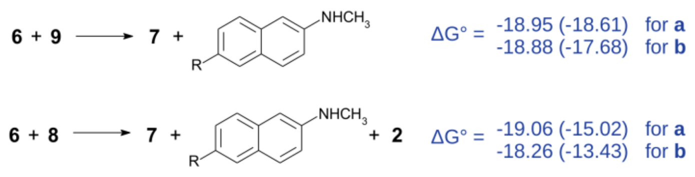

Scheme S2. Comparison between the thermochemistry with uncorrected CAM-B3LYP and with CAM-B3LYP-GD3BJ (in parenthesis). As main text Scheme 9.

3D views of some key stationary points

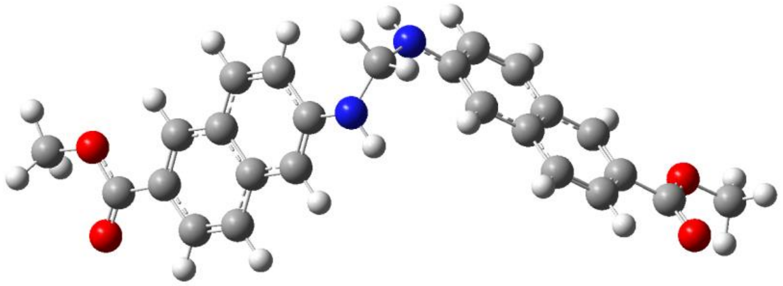

Figure S1. Structure of aminal **8b**

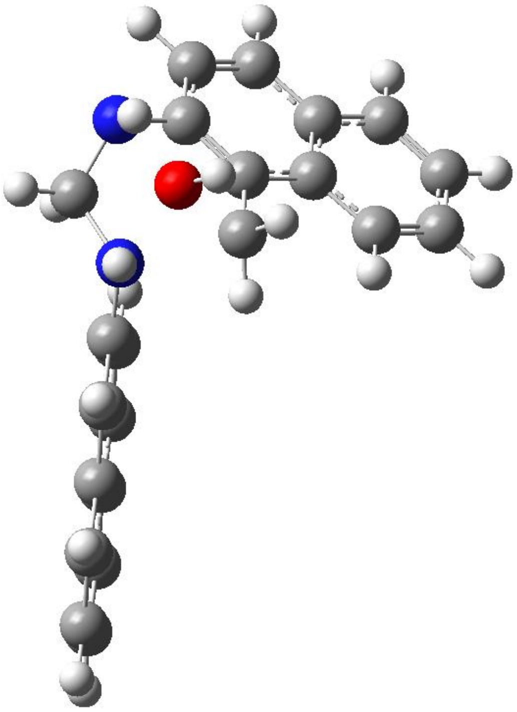

Figure S2. Structure of the transition state **TS-i2-6a**

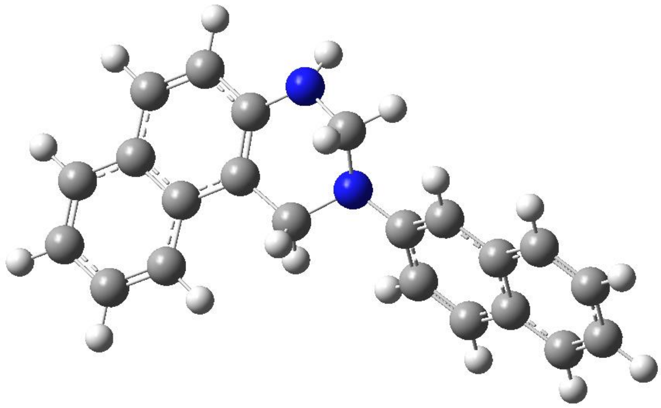

Figure S3. Structure of quinazoline **6a**

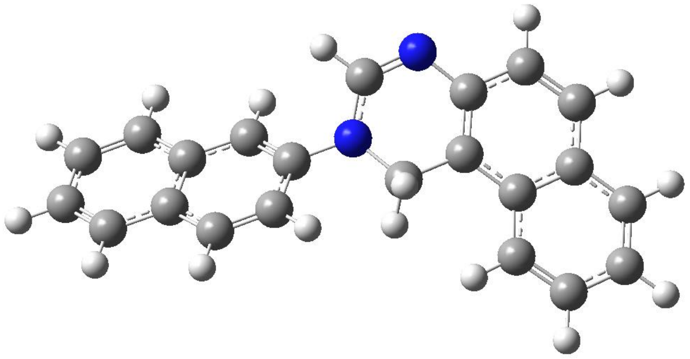

Figure S4. Structure of dihydroquinazoline **7a**

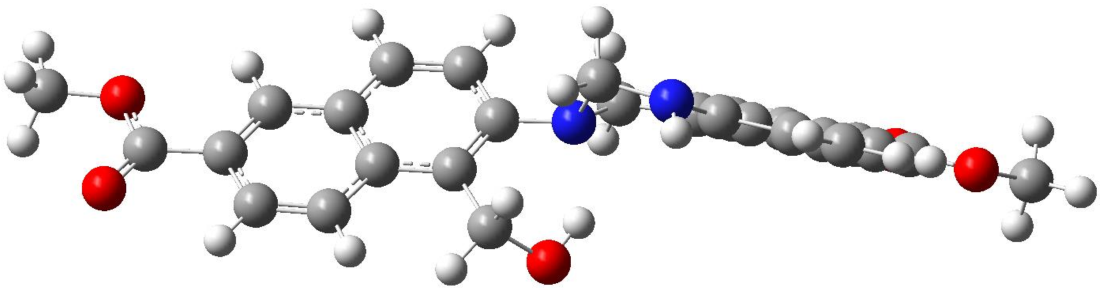

Figure S5. Structure of intermediate **i3b**

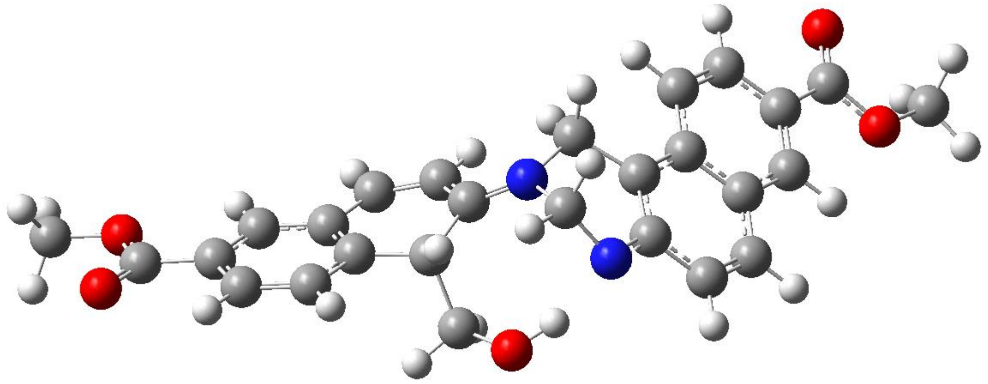

Figure S6. Structure of the tautomer of **i3b$_{tau}$**

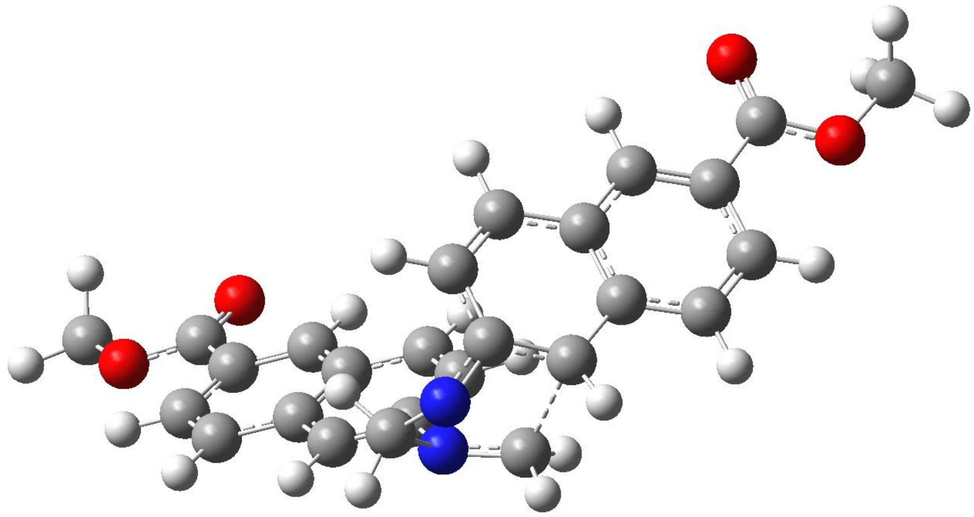

Figure S7. Structure of the transition state **TS-9-6b$_{tau}$**

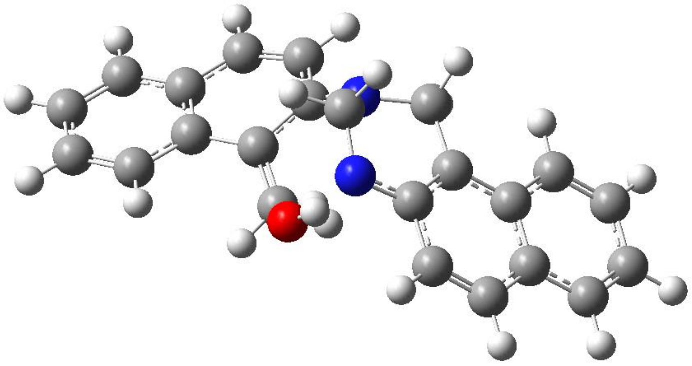

Figure S8. Structure of the transition state **TS-i3-3a**

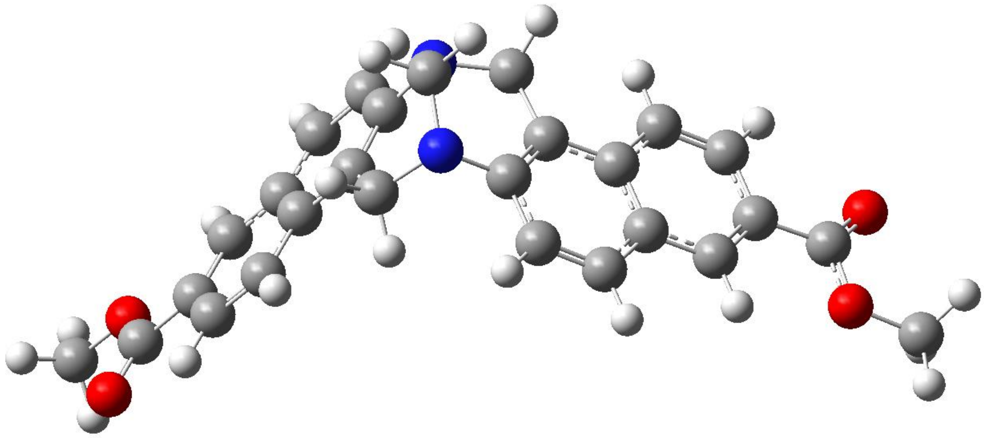

Figure S9. Structure of TB **3b**

^{1}H and ^{13}C NMR spectra of pure quinazoline **6a**

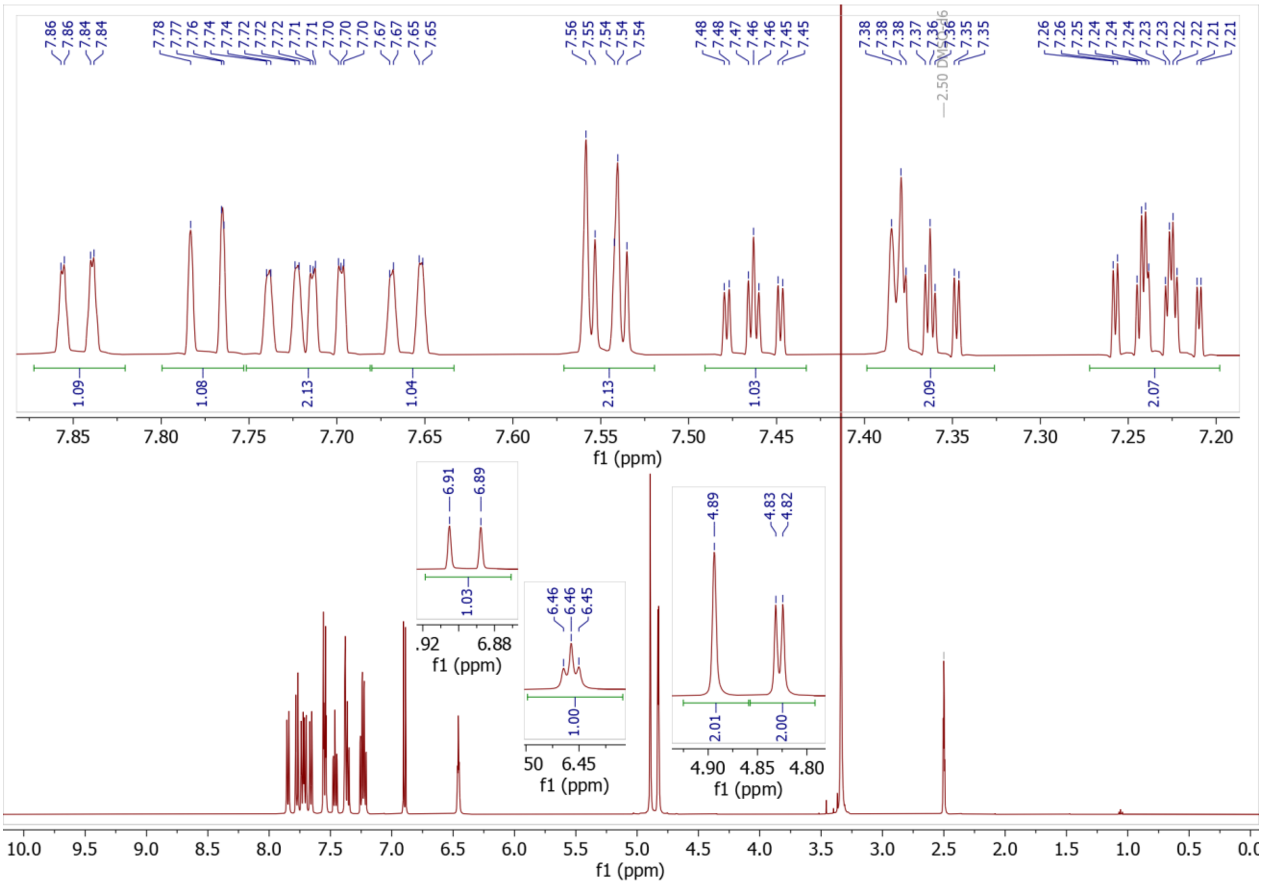

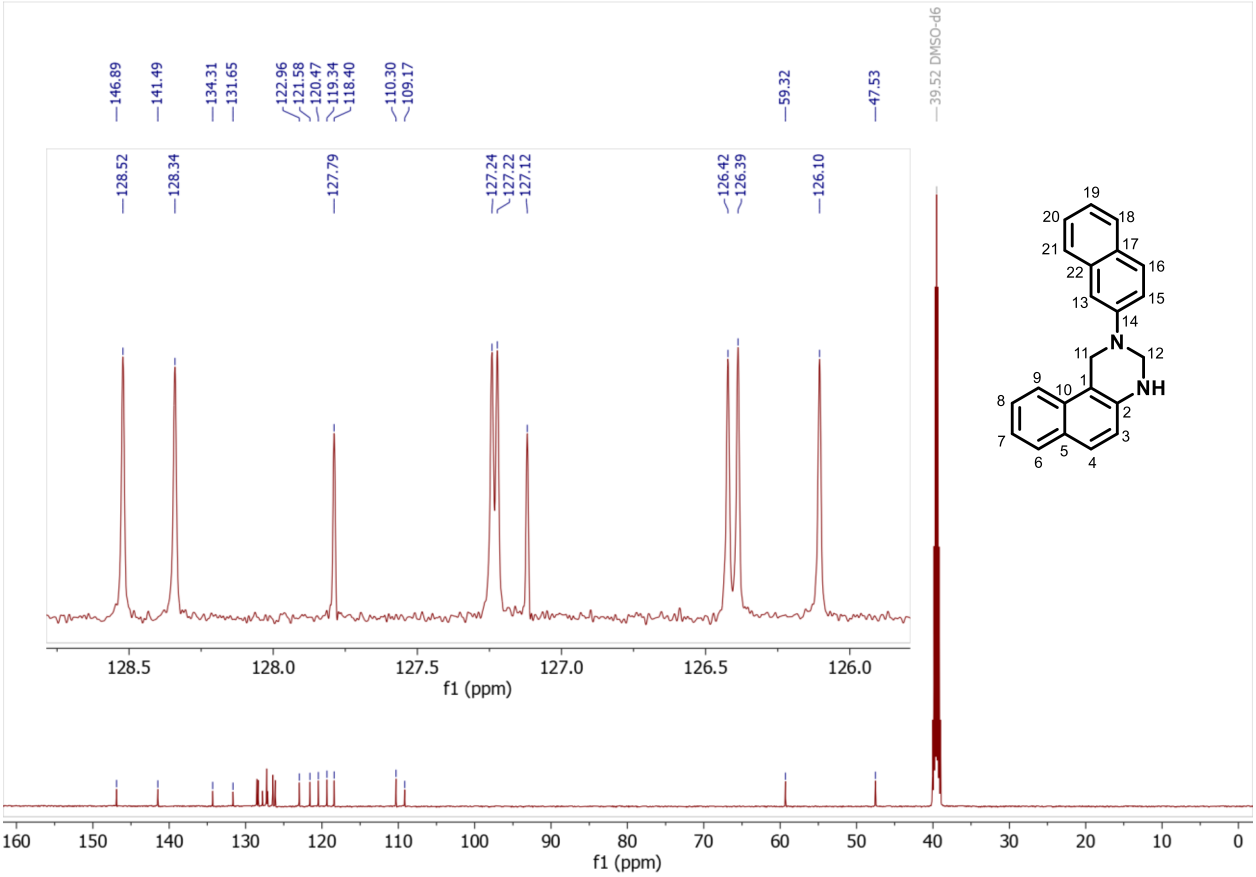

^{1}H and ^{13}C NMR spectra of pure bisquinazoline **16a**

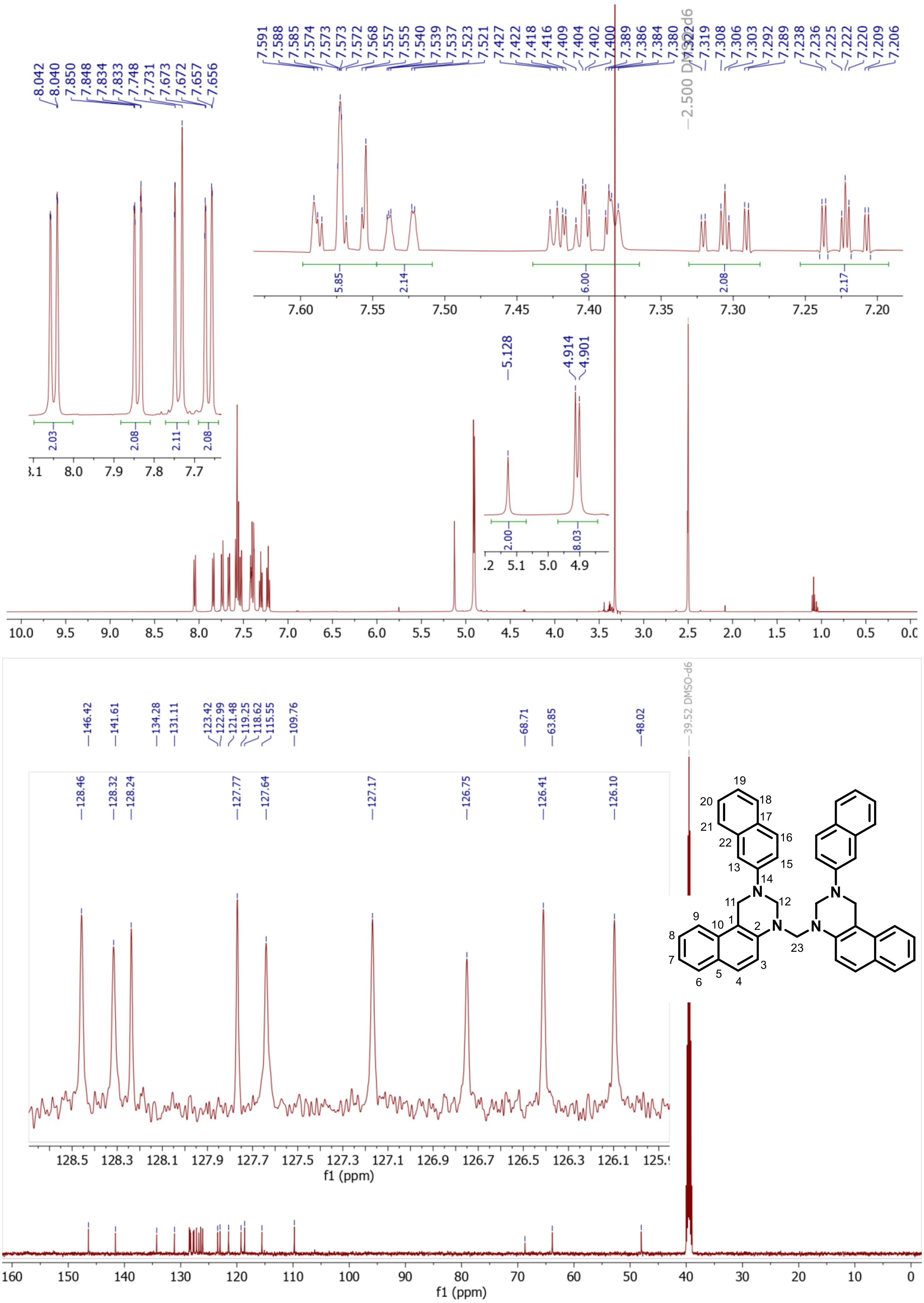

^{1}H NMR spectra of crude **8b** in DMSO-d_6 at various temperature

(the intensity normalized on the CH_2 signal of **8b**)

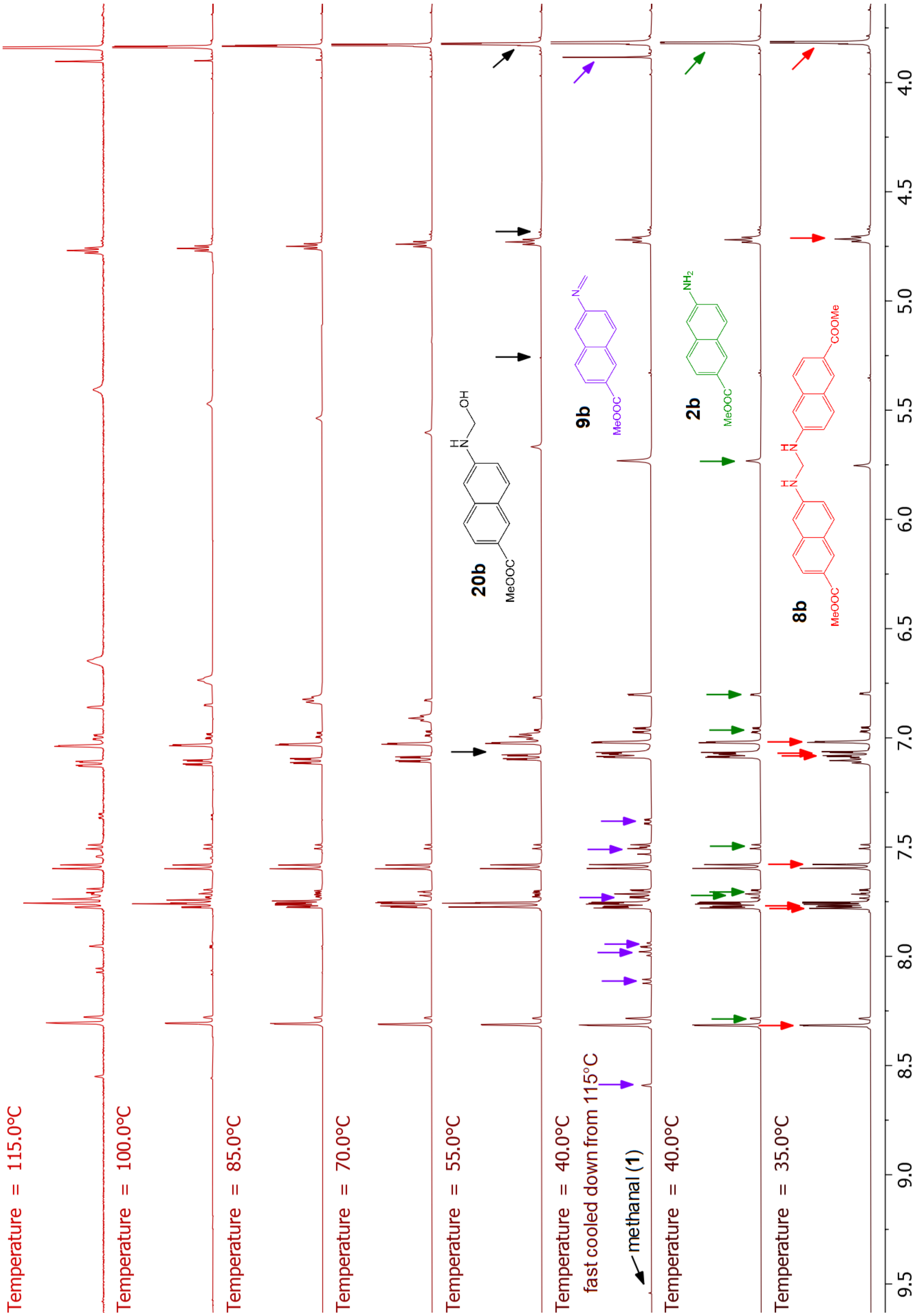

^{1}H-^{1}H DQF-COSY 2D NMR spectrum of **1**, **2b**, **8b**, **9b**, and **20b** mixture in DMSO-d_6 at 40°C

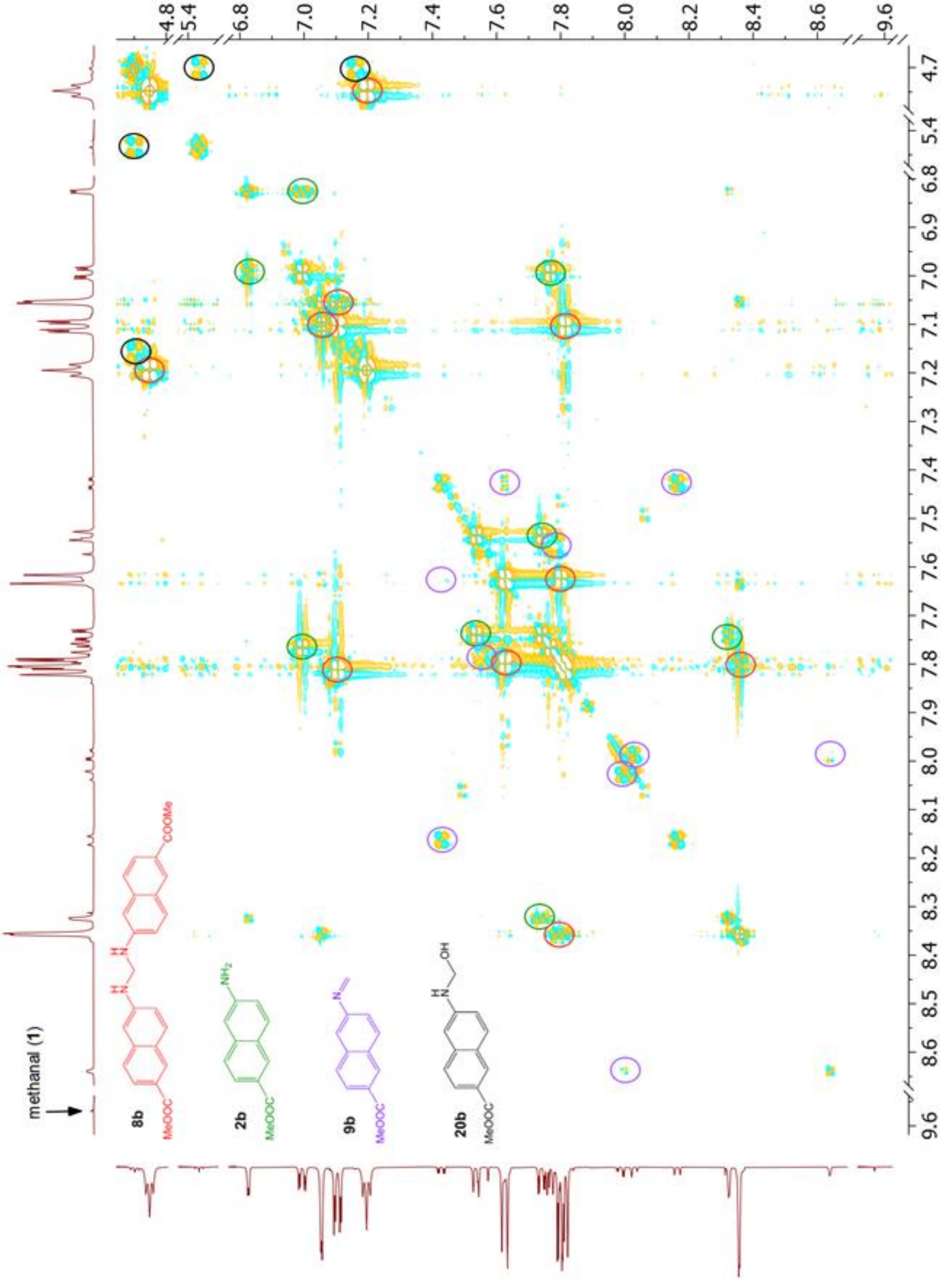

^{1}H-^{13}C HSQC and HMBC 2D NMR spectra of **1**, **2b**, **8b**, **9b**, and **20b** mixture in DMSO-d_6 at 40°C

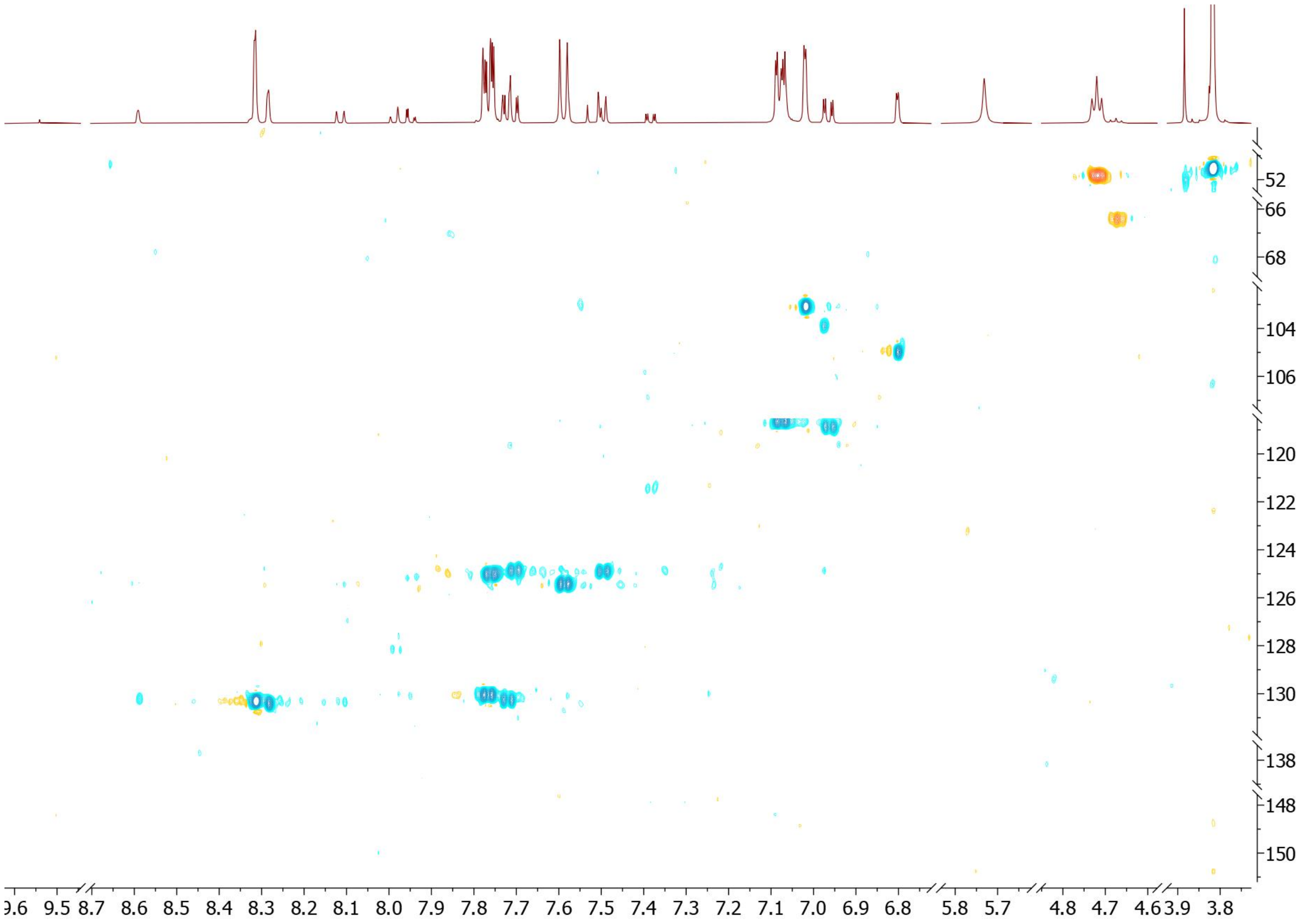

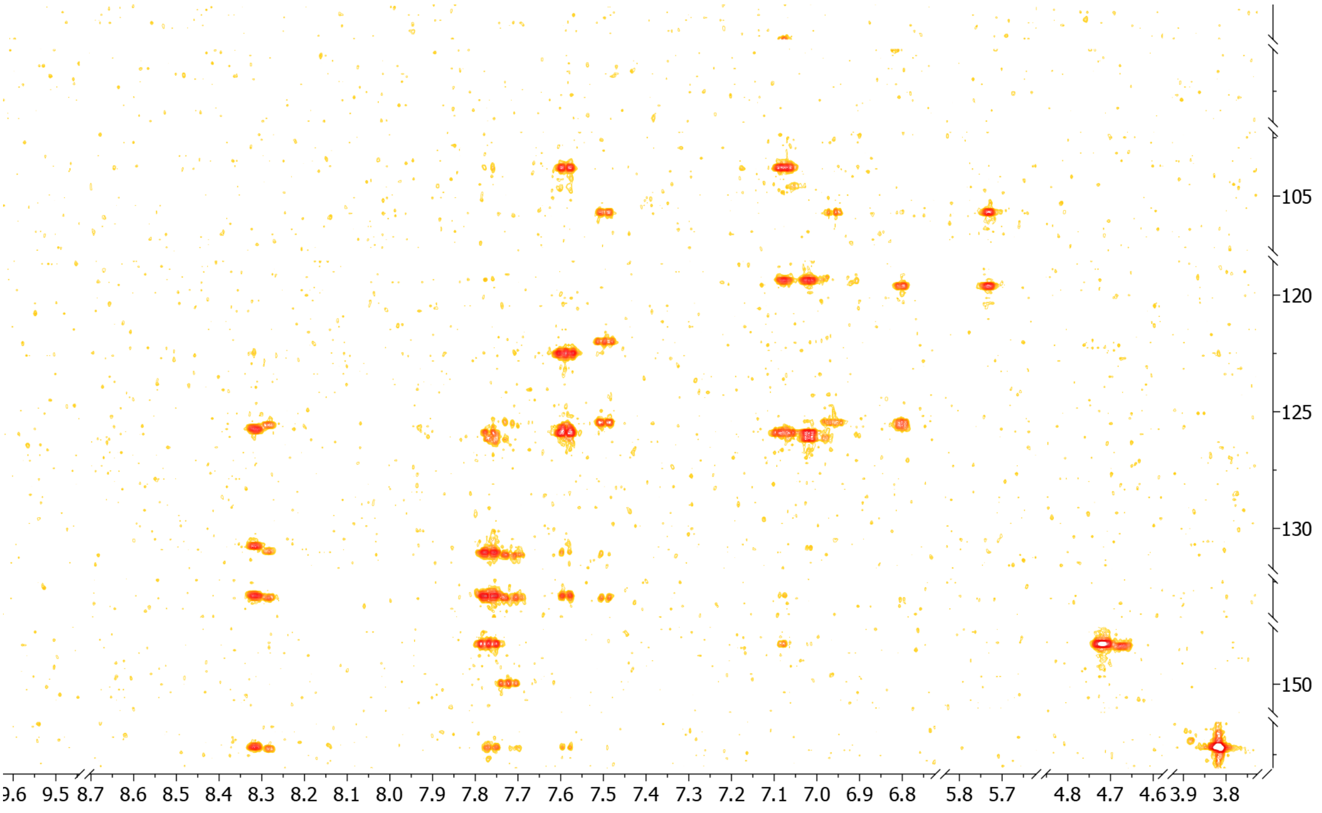

^{1}H NMR spectrum of formalin in DMSO-d_6 at 25°C

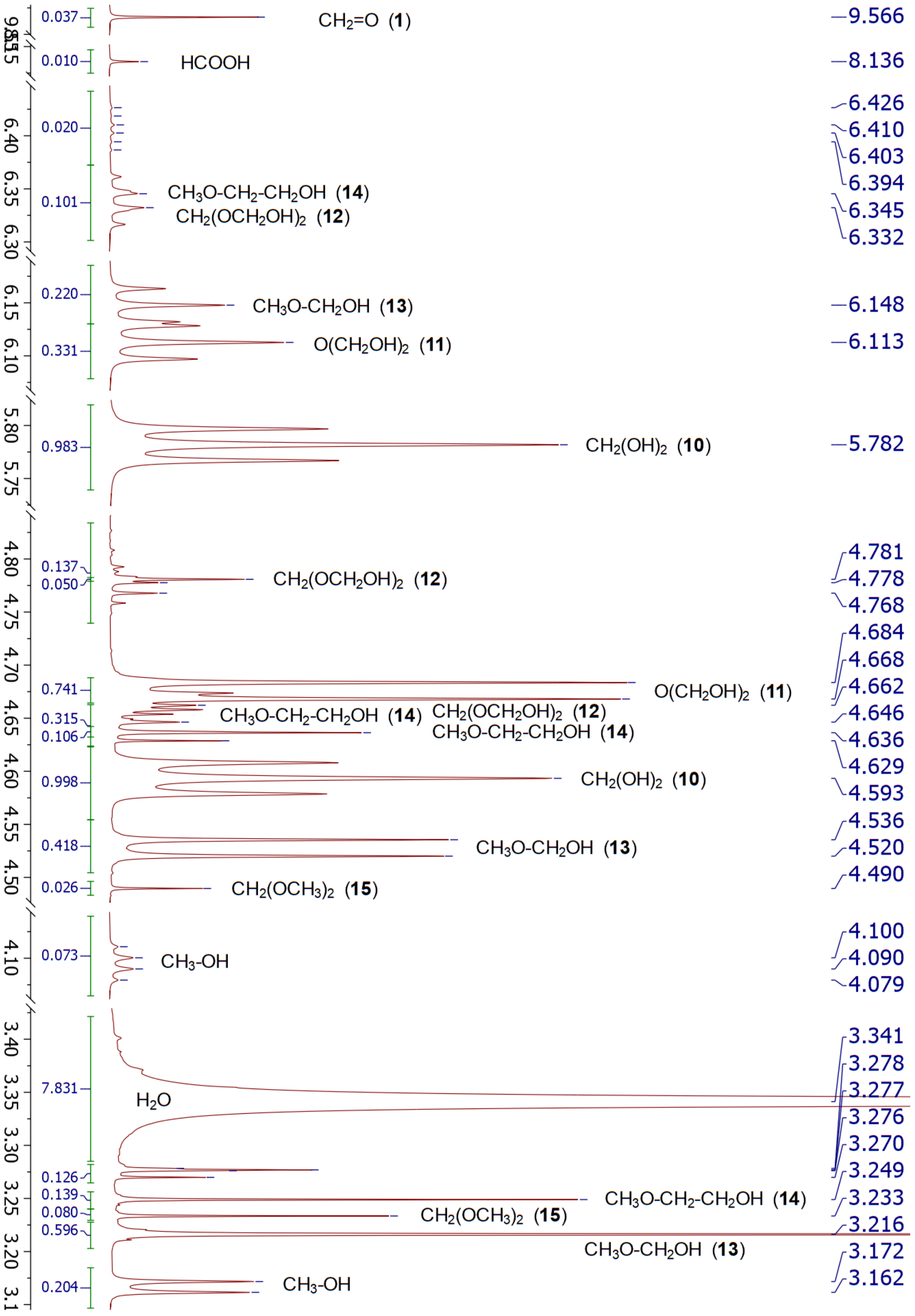

^{1}H-^{13}C HSQC and HMBC 2D NMR spectra of formalin in DMSO-d_6 at 25°C

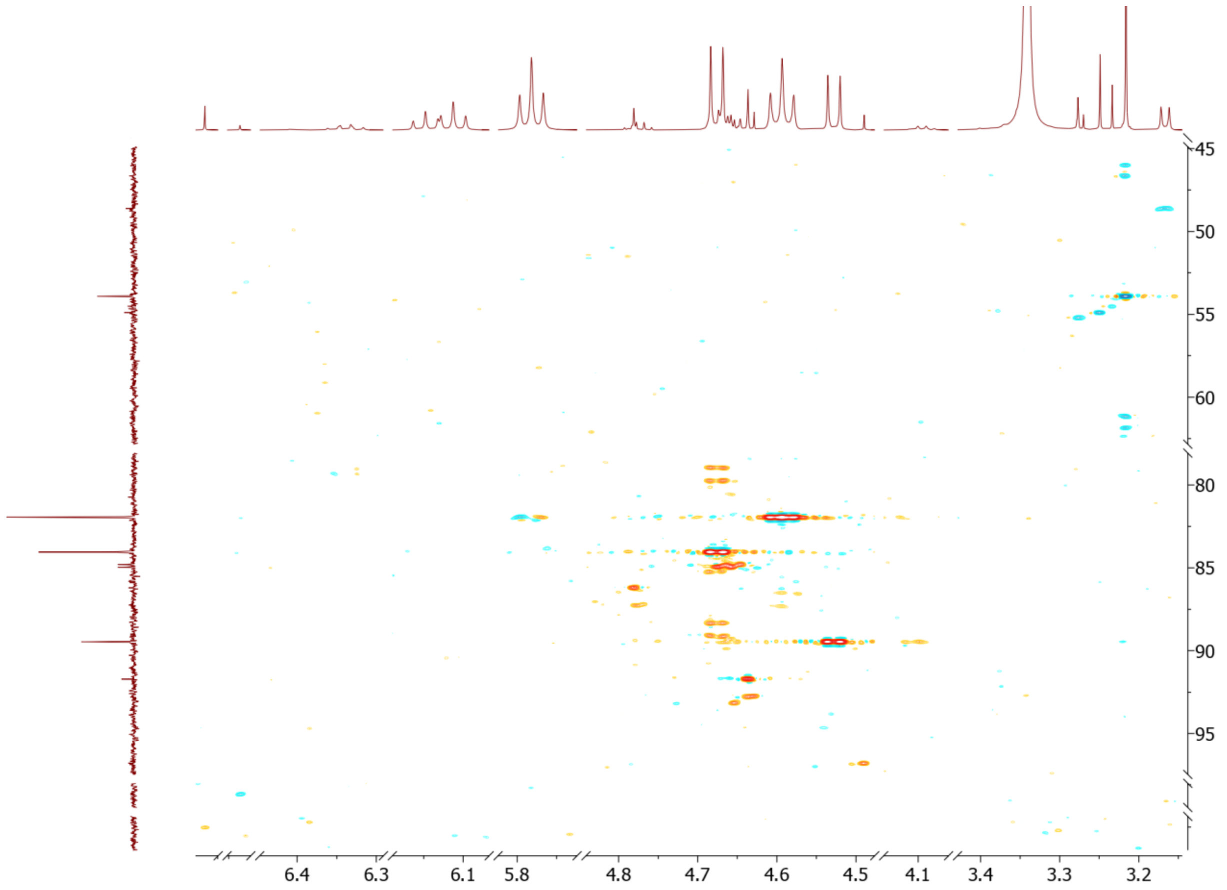

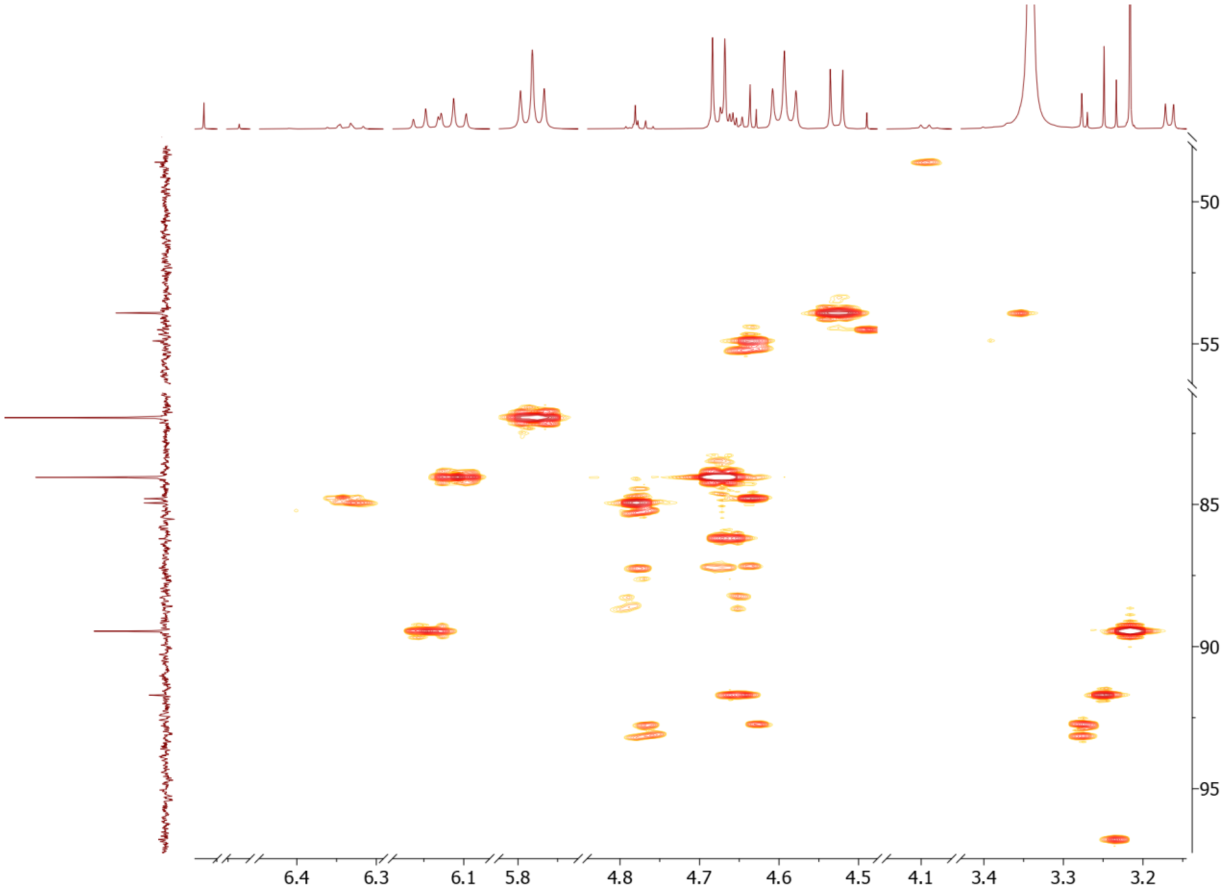

Supplement: Supplementary file 1 [file molecules-28-01549-s001.zip › molecules-2166615-supplementary.pdf]
